# Live and Heat-Inactivated *Lactiplantibacillus plantarum* Ameliorate Loperamide-Induced Constipation in Mice via Modulating Gut Microbiota, Short-Chain Fatty Acids and Gastrointestinal Function

**DOI:** 10.3390/nu18111658

**Published:** 2026-05-22

**Authors:** Hanlu Li, Xiaomin Feng, Feiliang Zhong, Xuegang Luo

**Affiliations:** Key Laboratory of Industrial Fermentation Microbiology of the Ministry of Education, College of Biotechnology, Tianjin University of Science and Technology, Tianjin 300457, China; lihanluha@163.com (H.L.); fxmxka@163.com (X.F.)

**Keywords:** *Lactiplantibacillus plantarum*, probiotics, constipation, gut microbiota, SCFAs

## Abstract

Aims: The effects of two *Lactiplantibacillus plantarum* strains and their probiotics on loperamide-induced constipation in mice were compared, and the possible mechanisms of the two strains in alleviating constipation were explored. Methods: KM mice were divided into the normal group, model group, positive control group, LTJ53 group, LP11824 group, HK-LTJ53 group and HK-LP11824 group. Loperamide was used to induce constipation in the mice. The study examined changes in defecation time, intestinal propulsion rate, gastric emptying rate, gastrointestinal peptides, colon histology, expression of intestinal barrier function genes, gut microbiota, and short-chain fatty acids (SCFAs). Results: Both live and postbiotic forms of *L. plantarum* significantly shortened defecation time, improved gastric emptying and intestinal motility, increased the levels of 5-hydroxytryptamine (5-HT), gastrin (GAS) and motilin (MTL), decreased the level of vasoactive intestinal peptide (VIP), restored colon morphology, upregulated the expression of Zonula Occludens-1 (ZO-1), mucin 2 (MUC2) and aryl hydrocarbon receptor (AhR), and downregulated the expression of aquaporin 4 (AQP4). They can also regulate the composition of the gut microbiota and alter the levels of SCFAs. Strain-specific effects were observed: LTJ53 was more effective in improving weight loss and gastric emptying, while LP11824 showed stronger efficacy in promoting small intestinal motility. Conclusions: *L. plantarum* and its postbiotics can relieve constipation through regulating the intestinal flora, enhancing gastrointestinal motility, adjusting the levels of neurotransmitters, and improving the intestinal barrier function. The specific effects of the two strains can support the selection of function-oriented precise intervention.

## 1. Introduction

The global prevalence of constipation is approximately 10–15%, with women, the elderly, and those with low dietary fiber intake at higher risk. Its prevalence is affected by a variety of factors such as region, lifestyle, and disease [1]. The incidence rate in developed countries is usually higher than in developing countries. Chronic constipation may have a long-term impact on quality of life and needs to be improved by a combination of dietary adjustments and medical interventions. While traditional drug treatments (such as stimulants or osmotic laxatives) can quickly relieve symptoms, long-term use can lead to adverse effects such as dependence and electrolyte imbalance, limiting long-term efficacy and highlighting the urgent need for safer treatment alternatives [2]. Probiotics, with their multi-target regulatory effects, offer a new, non-drug-dependent option for constipation management. Their core mechanism mainly revolves around the reconstruction of the gut microbiota and the bidirectional regulation of the gut–brain axis [3]. On the one hand, probiotics ferment dietary fiber to produce SCFAs, lowering the intestinal pH, directly stimulating intestinal peristalsis and inhibiting the growth of putrefactive bacteria [4]. On the other hand, probiotics play a key role in regulating the gut–brain axis. Specific strains can produce neuroactive substances such as γ-aminobutyric acid (GABA) and serotonin (5-HT) [5].

Clinical studies have confirmed that specific strains of *L. plantarum* can effectively shorten colonic transit time, increase defecation frequency, and soften stool, and usually require continuous intake for 1–4 weeks to show results [6]. Compared with chemical drugs, probiotics have significant safety advantages: they do not stimulate the intestinal nerves, have no drug dependence, and only occasionally cause mild abdominal bloating in the early stages [7]. They are especially suitable for patients with mild to moderate constipation, the elderly, and for the recovery of antibiotic-associated intestinal function.

However, probiotics are not emergency medications, and their effects are strain-specific, often requiring synergistic effects with prebiotics such as dietary fiber to achieve optimal efficacy [8]. In summary, probiotics provide a physiologically sound natural intervention strategy for functional constipation by reshaping the gut microbiota balance and regulating the gut–brain axis signaling network [9,10]. However, their clinical application still needs to be based on evidence and combined with lifestyle adjustments to achieve the best therapeutic effect.

This study mainly compared the effects of two strains of *L. plantarum* and their postbiotics on alleviating loperamide-induced constipation in mice. By systematically evaluating defecation time, gastrointestinal propulsion rate, levels of gastrointestinal bioactive peptides (5-HT, GAS, MTL, VIP), colonic tissue morphology, expression of barrier function-related genes, gut microbiota composition, and short-chain fatty acid content in mice, this study analyzed the correlation between gut microbiota and metabolic indicators and SCFAs. The aim was to reveal the efficacy and specificity of different bacterial strains (LTJ53 and 11824) and their postbiotics in regulating gut microbiota, gut–brain axis signaling, and gastrointestinal motility, providing experimental evidence for postbiotics as a safer and more stable constipation intervention strategy.

## 2. Materials and Methods

### 2.1. Experimental Strain Culture and Postbiotic Preparation

*L. plantarum* LTJ53 and 11824 were deposited in our laboratory. The collected bacterial suspension (live) was adjusted to 1 × 10^9^ CFU/mL for live bacterial interventions (LTJ53 and LP11824 groups). For postbiotic preparation, the live suspension (1 × 10^9^ CFU/mL) was heat-inactivated at 100 °C for 30 min, and complete inactivation was confirmed by plating on MRS agar (no colony growth after 72 h). The inactivated suspension was then concentrated to a final density of 2 × 10^9^ cells/mL (based on microscopic cell counting, equivalent to the viable count before inactivation). This heat-inactivated preparation is referred to as ‘postbiotics’ or ‘heat-inactivated postbiotics’ throughout the manuscript.

In this study, “postbiotics” is used as the general term for the heat-inactivated bacterial preparation. “Heat-inactivated postbiotics” (abbreviated as HK-) is used interchangeably to emphasize the inactivation method (heat-killed). Both terms refer to the same preparation: whole, non-viable *L. plantarum* cells (100 °C for 30 min), without lysis or fractionation. The key distinction from live probiotics is the absence of colony-forming ability, confirmed by plating on MRS agar (no growth after 72 h at 37 °C under anaerobic conditions).

### 2.2. Experimental Animals and Groups

Sample size was estimated based on a power analysis using G*Power software (version 3.1.9.7). Assuming a large effect size (Cohen’s d = 1.2) for the primary outcome (first black stool time), with α = 0.05 and power (1 − β) = 0.80, the calculated minimum sample size was 5 mice per group. To account for potential dropouts or technical failures during intestinal motility measurements, we included 6 mice per group (*n* = 6).

A total of 42 healthy male specific pathogen-free (SPF) KM mice (5 weeks old) were purchased from the Institute of Laboratory Animal Science, Chinese Academy of Medical Sciences. The KM mice were placed in an animal laboratory at 24 °C with a controlled day-night cycle of 12 h. Mice were given free access to water and food. After 7 days of acclimatization, they were randomly divided into 7 groups and began gavage intervention. The experimental groups included a normal control group (NC) receiving sterile saline via gavage; a constipation model group (M) receiving loperamide hydrochloride via gavage, followed by sterile saline 1 h later; a positive control group (PC) receiving loperamide hydrochloride via gavage, followed by 2 × 10^9^ CFU/mL of *Bacillus subtilis* dual live bacteria granules via gavage 1 h later; the *L. plantarum* LTJ53 intervention group (LTJ53) and the *L. plantarum* 11824 intervention group (LP11824) receiving loperamide hydrochloride via gavage, followed by 1 × 10^9^ CFU/mL bacterial suspension via gavage 1 h later; and the heat-inactivated *L. plantarum* LTJ53 postbiotic intervention group (HK-LTJ53) and the *L. plantarum* 11824 postbiotic intervention group (HK-LP11824) receiving loperamide hydrochloride via gavage, followed by 2 × 10^9^ CFU/mL bacterial suspension via gavage 1 h later. All groups had free access to food and water, and the mice’s weight was recorded daily during the experiment. All animal procedures were conducted following the ARRIVE guidelines (https://www.nc3rs.org.uk/arrive-guidelines accessed on 3 May 2024) and approved by the Academic Committee of Tianjin University of Science and Technology (KJDA20240114). These guidelines strictly adhered to the ARRIVE principles.

### 2.3. Determination of the First Black Stool Time

On the last day of gavage administration, the time of the first black stool in mice was determined. The method was as follows: Except for the NC group, mice in other groups were administered loperamide hydrochloride (10 mg/kg) (0.25 mL) by gavage. One hour later, all experimental groups were administered ink containing their respective gavage contents. The time of the first black stool in each animal was recorded from the start of the ink administration. The time of the first black stool in the last mouse of group M was used as the end time, and the differences in the time of black stool between each treatment group and group M were compared.

### 2.4. Determination of Small Intestinal Propulsion Rate

Mice were fasted for 16 h. Each mouse was administered 0.4 mL of semi-solid nutrient paste by gavage 30 min before ocular blood collection. The mice were euthanized by cervical dislocation 30 min later and dissected. The small intestine (from the pylorus to the terminal ileum) was removed and placed flat on the experimental table. The total length of the small intestine and the distance from the pylorus to the nutrient paste (propulsion length) were recorded. The propulsion rate was calculated using the following formula.

Small intestine propulsion rate (%) = Ink propulsion length (cm)/Total small intestine length (cm) × 100%.

### 2.5. Determination of Gastric Emptying Rate

Mice were fasted for 16 h with free access to water. Preparation of semi-solid nutrient paste: A total of 5 g of sodium carboxymethyl cellulose was dissolved thoroughly in 125 mL of deionized water. Subsequently, 8 g of skim milk powder, 4 g of soluble starch, 4 g of sucrose, and 2 mL of black ink were added and homogenized. The mixture was diluted 1:1 with water prior to use. Prior to gavage, the actual mass of 0.4 mL of the nutrient paste administered to each mouse was determined using an electronic analytical balance (mean value of three calibrations, approximately 0.42 ± 0.01 g), denoted as *Winitial*. Thirty minutes post-gavage, mice were euthanized, and the stomachs were excised intact. The external surface of the stomach was rinsed with physiological saline, blotted dry with filter paper, and weighed (stomach plus residual contents), recorded as *Wtotal*. The stomach was then incised along the greater curvature, and all gastric contents were gently removed. The gastric wall was rinsed with physiological saline, blotted dry, and weighed (net stomach weight), denoted as *Wstomach*. The mass of residual gastric contents (*Wresidual*) was calculated as *Wtotal* − *Wstomach*. Gastric emptying rate (%) was computed using the formula:

[(*Winitial* − *Wresidual*)/*Winitial*] × 100%.

### 2.6. Detection of Constipation-Related Indicators Such as Gastrointestinal Active Peptides

Gastric tissue was rinsed with PBS and homogenized using a homogenizer (Jingxin, Shanghai, China). After thorough homogenization, the tissue was centrifuged at 12,000 r/min for 10 min, and the supernatant was collected. The standard curves and sample determinations for GAS (H239-1-2, Nanjing Jiancheng, Nanjing, China), MTL (H182-1-2, Nanjing Jiancheng, Nanjing, China), VIP (H219-1-2, Nanjing Jiancheng, Nanjing, China), and 5-HT (H104-1-2, Nanjing Jiancheng, Nanjing, China) were performed according to the ELISA kit instructions. The concentration of the analytes in the sample was calculated based on the absorbance.

### 2.7. Histopathological Examination of Colonic

At the end of the experiment, the mouse colon was extracted. The intestinal mucosa tissue was isolated, dehydrated using a fully enclosed tissue dehydrator (Leica Biosystems, Nussloch, Germany) treated with xylene for transparency, embedded in a biological tissue embedding machine (Leica Biosystems, Nussloch, Germany), and sliced using a rotary slicer for routine pathology sections (Leica Biosystems, Nussloch, Germany). Subsequently, dewaxing and hydration procedures were conducted, hematoxylin and eosin (HE) staining was performed using the kit (C0105S, Biyuntian, Shanghai, China) (consult the manual for specific instructions), and the pathological morphology of the mouse intestinal mucosa was observed under a microscope (Olympus, Tokyo, Japan).

### 2.8. RT-PCR

The sequences of GAPDH (Glyceraldehyde-3-phosphate dehydrogenase), AQP4, AhR, ZO-1 and MUC2 published in GenBank were used as templates to design primers (Table 1). The RT-PCR reaction was performed at 95 °C for 5 min, 95 °C for 30 s, at an annealing temperature of 54–58 °C for 30 s, at 72 °C for 30 s, with 30 cycles, and at 72 °C for 10 min.

### 2.9. 16s DNA Sequencing

Genomic DNA was extracted from mouse colon contents using the CTAB/SDS method. Briefly, 200 mg of colon contents were mixed with 1 mL CTAB/SDS lysis buffer (containing 2% CTAB, 1% SDS, 100 mM Tris-HCl pH 8.0, 20 mM EDTA, 1.4 M NaCl), ground in liquid nitrogen, and incubated at 65 °C for 1 h. After extraction with chloroform–isoamyl alcohol (24:1), DNA was precipitated with isopropanol and washed with 70% ethanol. DNA purity and concentration were assessed using a NanoDrop 2000 spectrophotometer (Thermo Fisher Scientific, Wilmington, USA), and integrity was verified via 1% agarose gel electrophoresis. The V3–V4 hypervariable region of the 16S rRNA gene (16S rDNA) was PCR-amplified using universal bacterial primers 338F (5′-ACTCCTACGGGAGGCAGCA-3′) and 806R (5′-GGACTACHVGGGTWTCTAAT-3′), and microbiome diversity was analyzed using Kruskal–Wallis test with Dunn’s post hoc test. Principal component analysis (PCoA) and linear discriminant analysis effect size (LEfSe) were used to analyze beta diversity and significant differences in microbial communities. Sequencing was performed using the Illumina NovaSeq 6000 platform.

### 2.10. Statistical Analysis

All quantitative data are expressed as mean ± standard error of the mean (SEM). Intergroup differences were evaluated by one-way analysis of variance (ANOVA) for multiple comparisons using GraphPad Prism v9.0.0. Statistical significance thresholds were defined as follows: ns (not significant) for *p* > 0.05; * *p* < 0.05; ** *p* < 0.01; *** *p* < 0.001. Quantitative data are presented as mean ± standard deviation (SD) from three independent biological replicates.

## 3. Results

### 3.1. L. plantarum and Its Postbiotics Improve Physiological Indicators Related to Constipation in Mice

**Figure 1:**
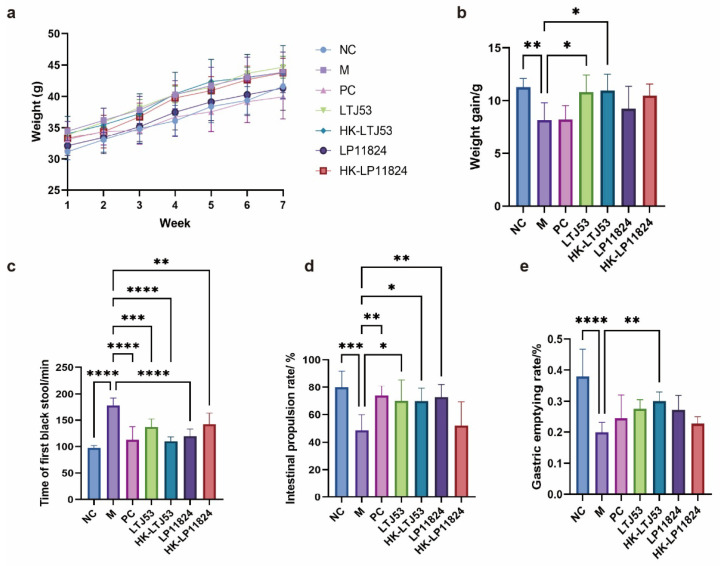
Effects on physiological indicators of constipated mice. (**a**) Trend of mouse body weight change. (**b**) Change in mouse body weight. (**c**) Time to first black stool in mice. (**d**) Mouse small intestinal propulsion rate. (**e**) Gastric emptying rate in mice. Statistical significance: * *p* < 0.05, ** *p* < 0.01, *** *p* < 0.001, **** *p* < 0.0001 vs. M group, one-way ANOVA with Tukey’s post hoc test, n = 6.

To evaluate the effects of *L. plantarum* and its postbiotics on weight changes in constipated mice, we weighed each mouse, as shown in Figure 1a. We calculated the weight gain in each group, as shown in Figure 1b. The weight gain rate in the M group was significantly lower than that in the NC group. This indicates a positive correlation between loperamide-induced constipation and slower weight gain in mice, presumably due to loss of appetite in the model mice. Treatment with *L. plantarum* LTJ53 and its postbiotics via gavage can alleviate this phenomenon. However, the figure shows that the positive control drug and treatment with *L. plantarum* 11824 and its postbiotic via gavage did not significantly improve weight loss in constipated mice. The time it takes for a mouse to expel its first black feces is an important indicator of intestinal motility in constipated mice. The longer the time it takes for the first black feces to be expelled, the more severe the constipation [11]. By administering activated charcoal semi-solid nutritional paste by gavage, the peristaltic position of digested food in the mouse intestine and the time of expulsion of the first black feces can be marked. Therefore, it can be used to evaluate the effects of *L. plantarum* and postbiotics on defecation in constipation model mice. The experimental results are shown in Figure 1c.

The time to first black stool in group M mice was significantly higher than that in group NC and treatment group, which indicates that gavage administration of *L. plantarum* and its postbiotics can accelerate intestinal motility in mice, reduce defecation time, and thus relieve constipation. Small intestinal propulsion rate can directly reflect the peristaltic ability of the small intestine. As shown in Figure 1d, there is a significant difference in small intestinal propulsion rate between the model group and the NC group (*p* < 0.001), indicating that loperamide can cause a decrease in the peristaltic ability of the small intestine in mice. By observing the length of ink propulsion in the small intestine, it can be seen that all treatment groups can promote small intestinal motility. However, the positive control drugs and *L. plantarum* 11824 showed better effects in promoting small intestinal peristalsis (*p* = 0.0053, 0.0086 < 0.01), which can reduce the digestion time of food in the small intestine, accelerate the propulsion of ink in the small intestine, and improve constipation symptoms in mice. Gastric emptying rate refers to the ratio of the mass of the same mass of semi-solid nutritional paste administered by gavage to the amount of food remaining in the stomach within a certain period of time. As shown in Figure 1e, compared with the NC group, the gastric emptying rate of mice in the M group was reduced (*p* < 0.0001), indicating that loperamide-induced constipation leads to a decrease in gastric motility and emptying capacity. The HK-LTJ53 group significantly improved the gastric emptying ability of constipated mice (*p* = 0.0085 < 0.01). The above results indicate that the positive control drug and *L. plantarum* and its postbiotics can improve the relevant physiological indicators of constipated mice. However, *L. plantarum* LTJ53 and its postbiotics seem to be more effective in improving weight loss and gastric emptying. The positive control drug and *L. plantarum* 11824 showed better effects in promoting small intestinal motility.

### 3.2. L. plantarum and Its Postbiotics Improve Biochemical Indicators in Constipated Mice

In the central nervous system, there are some bioactive substances that are originally secreted in the gastrointestinal tract. They also play an important role in the brain and are therefore known as brain–gut peptides or neurotransmitters [12]. These substances form a complex communication network between the brain and the gastrointestinal tract, and by acting on specific receptors in the nervous system, they participate in the regulation of a variety of physiological processes, including mood, appetite, metabolism, and pain perception [13,14]. This two-way communication mechanism reveals the close connection between the digestive system and the brain, which is of great significance for our understanding of the interaction between brain function and the digestive system [15]. In the gastrointestinal tract, 5-HT, as a gastrointestinal hormone, is involved in intestinal motility and secretion. It can stimulate the contraction of intestinal smooth muscle and promote intestinal peristalsis, thereby aiding in the digestion and absorption of food [16].

**Figure 2:**
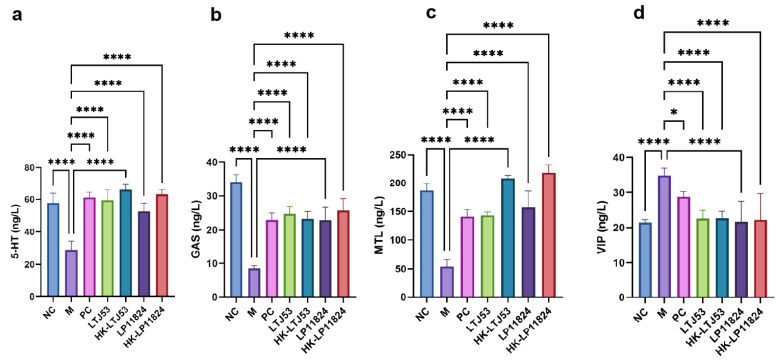
Effects on relevant biochemical indicators in constipated mice. (**a**–**d**) ELISA detection of 5-HT, GAS, MTL, and VIP. Statistical significance: * *p* < 0.05, **** *p* < 0.0001 vs. M group, one-way ANOVA with Tukey’s post hoc test, n = 6.

As shown in Figure 2a, 5-HT secretion was significantly reduced in the M group (*p* < 0.0001), while its content was significantly increased in each treatment group (*p* < 0.0001), and the HK-LTJ53 group showed a higher level than the NC group. GAS is an important gastrointestinal hormone, mainly secreted by G cells in the gastric antrum and duodenum. GAS can promote the contraction of smooth muscle in the gastrointestinal tract and accelerate gastric emptying, thereby propelling food into the small intestine [17]. As shown in Figure 2b, compared with the NC group, the gastrin secretion level of mice in the M group was reduced and the difference was extremely significant (*p* < 0.0001). Compared with the model group mice, the gastrin secretion level of other groups was significantly increased (*p* < 0.0001), indicating that the treatment group can effectively stimulate the secretion of gastrin to relieve constipation. The main physiological function of MTL is to promote gastrointestinal motility and accelerate gastric emptying [18]. It promotes defecation by stimulating the influx of calcium ions, regulating gastrointestinal motility, increasing gastric contractility, and promoting segmental movement of the small intestine [19]. As shown in Figure 2c, the secretion of motilin in group M was significantly reduced (*p* < 0.0001), while the secretion in other groups was significantly increased (*p* < 0.0001), thereby promoting and accelerating gastrointestinal motility. VIP is a major inhibitory neurotransmitter in the enteric nervous system. It can affect blood circulation and gastrointestinal motility by binding to its receptors, causing vasodilation and smooth muscle relaxation [20]. As shown in Figure 2d, compared with the NC group, the M group mice had significantly increased VIP secretion (*p* < 0.0001). Compared with the M group mice, the PC group had significantly lower secretion levels (*p* = 0.049 < 0.05), and the other groups had extremely significantly lower secretion levels (*p* < 0.0001). The results showed that gavage administration of *L. plantarum* and its postbiotics increased the levels of 5-HT, GAS, and MTL in mice with loperamide-induced constipation, inhibited VIP secretion, and thus accelerated gastrointestinal motility. Both strains of *L. plantarum* and their postbiotics exhibited favorable efficacy in improving the biochemical indices of constipated mice.

### 3.3. L. plantarum and Its Postbiotics Improve Colonic Tissue Morphology in Constipated Mice

**Figure 3:**
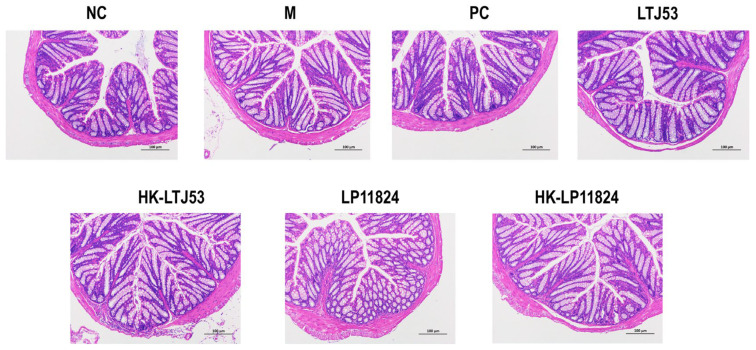
Effects on colon morphology in constipated mice.

To further investigate the effects of *L. plantarum* and its postbiotics on the colonic tissue of constipated mice, H&E staining was performed to observe the morphology of the colonic tissue. As shown in Figure 3, the intestinal wall of the NC group mice was thick and goblet cells were evenly distributed. Loperamide induces large gaps between incomplete intestinal villi and ruptures in colonic tissue, thus disrupting the integrity of the mucosal structure. Gavage administration of *L. plantarum* and its postbiotics restored the colonic wall cells of mice to a healthy state, and the morphological characteristics of the intestinal mucosa were restored to a certain extent, increasing the thickness of the colonic muscle layer and the number of goblet cells.

### 3.4. L. plantarum and Its Postbiotics Regulate the Expression of Metabolism-Related Genes in Constipated Mice

**Figure 4:**
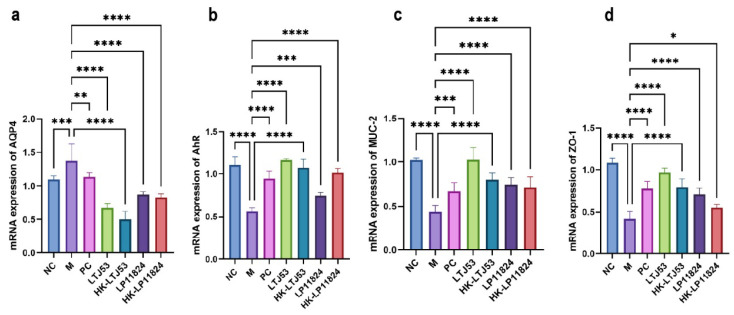
Effects on the expression of metabolism-related genes in constipated mice. (**a**–**d**) qPCR was used to detect the expression of AQP4, AhR, MUC2, and ZO-1 mRNA. Statistical significance: * *p* < 0.05, ** *p* < 0.01, *** *p* < 0.001, **** *p* < 0.0001 vs. M group, one-way ANOVA with Tukey’s post hoc test, n = 6.

AQP4 is an aquaporin mainly expressed in tissues such as the kidneys and gastrointestinal tract [21]. It is involved in regulating the permeability of water molecules on cell membranes, thereby affecting the balance of water and electrolytes [22]. As shown in Figure 4a, AQP4 mRNA expression was upregulated in the colon of constipated mice (*p* < 0.001), which may be related to excessive intestinal water absorption. *L. plantarum* and its postbiotics significantly downregulated AQP4 mRNA expression (*p* < 0.0001), thereby regulating the problem of disordered intestinal water absorption. As shown in Figure 4b, the mRNA expression level of the aryl hydrocarbon receptor AhR shows an opposite trend to that of AQP4. *L. plantarum* and its postbiotics may affect the function of the central nervous system by activating AhR, thereby relieving constipation. This effect may be related to AQP4-mediated water regulation. MUC2 is a major component of the intestinal mucus layer. It lubricates the intestines, reduces friction between feces and the intestinal wall, and thus helps feces pass through smoothly [23]. When constipated, if the secretion of MUC2 decreases, it may lead to insufficient intestinal lubrication, which can worsen constipation symptoms. As shown in Figure 4c, the expression level of MUC2 mRNA was significantly downregulated in group M, while the expression of its mRNA was significantly upregulated in other groups (*p* < 0.0001). ZO-1 is a tight junction protein that is mainly found at the tight junctions of epithelial cells and is essential for maintaining intercellular barrier function and selective permeability [24]. As shown in Figure 4d, the expression level of ZO-1 mRNA in the model group was significantly downregulated (*p* < 0.0001), while the expression level of ZO-1 mRNA in each treatment group was significantly upregulated (*p* < 0.0001). ZO-1 plays a crucial role in regulating the barrier function of intestinal epithelial cells, helping to prevent the penetration and spread of inflammatory mediators. Therefore, normal ZO-1 function may help reduce constipation-related intestinal inflammation.

### 3.5. L. plantarum and Its Postbiotic Balance in the Gut Microbiota of Constipated Mice

**Figure 5:**
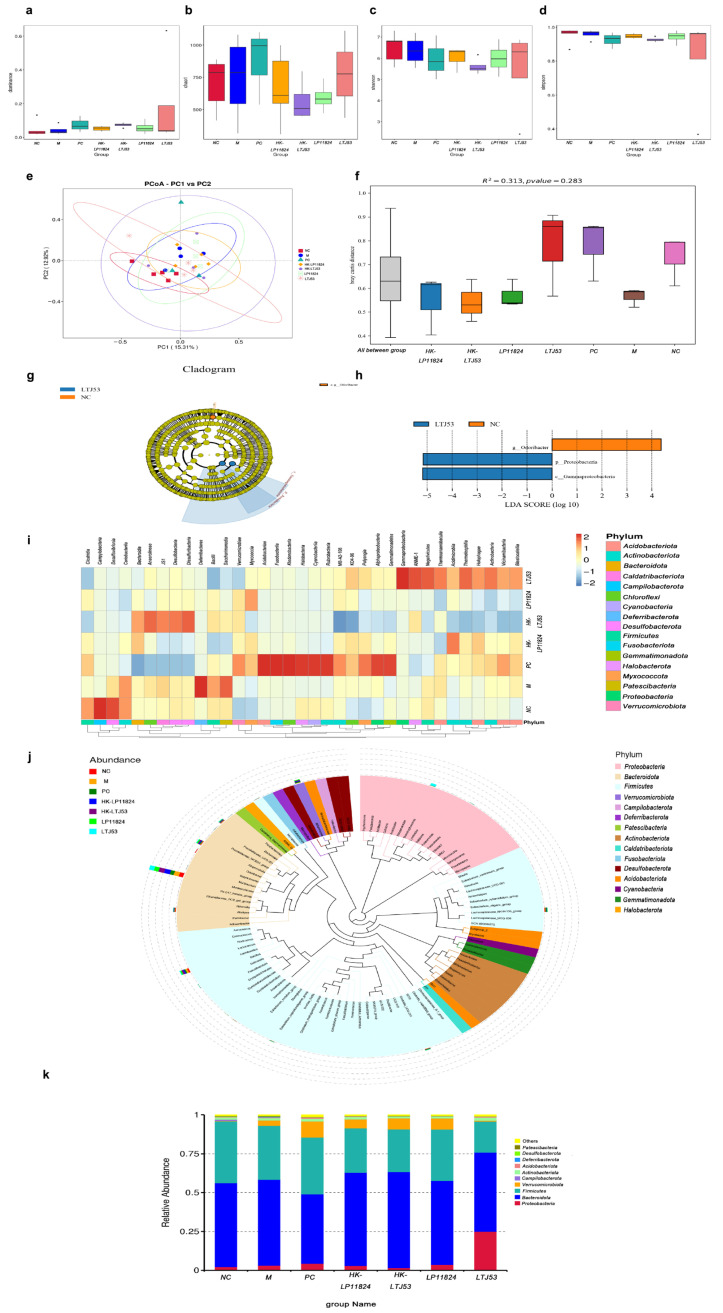
Effects on the gut microbiota of constipated mice. (**a**–**d**) Alpha diversity index (dominance, Chao1, Shannon, Simpson) difference analysis. (**e**) PCoA analysis. (**f**) Permanova analysis. (**g**,**h**) LEfSe analysis. (**i**) Species abundance clustering diagram (class level). (**j**) Phylogenetic tree constructed from representative sequences of species at the genus level. (**k**) Top 10 bar chart of relative abundance of species (phylum level). Kruskal–Wallis test with Dunn’s post hoc test, FDR-corrected.

In the intergroup difference analysis of the Alpha diversity index, box plots can intuitively reflect the median, dispersion, maximum, minimum, and outliers of species diversity within a group. As shown in Figure 5a–d, each group can increase the abundance of gut microbiota in constipated mice after intervention, with the most significant effect in the LTJ53 group. PCoA (Principal Coordinate Analysis) is a multivariate statistical method based on the distance matrix between samples, used to visualize differences between samples. As shown in Figure 5a, the NC group and the M group showed a certain separation trend on the PC1 axis, indicating that there were differences between the two groups in the direction of variation represented by PC1. PERMANOVA analysis revealed that the grouping factor explained 31.3% of the variation in the composition of the gut microbiota (R^2^ = 0.313). However, this effect did not reach statistical significance (pseudo-F = 1.82, *p* = 0.283, with 9999 permutations). This overall non-significance is likely due to the high intra-group individual variation (as shown in the PCoA plot) and the relatively small sample size. This indicates that loperamide-induced constipation does indeed cause measurable changes in the structure of the gut microbiota. LEfSe analysis (LDA score > 3.0, *p* < 0.05) identified differentially abundant bacterial taxa between the LTJ53 group and the NC group. Specifically, the LTJ53 group was enriched in *Gammaproteobacteria* and *Protebbacteria*, while the NC group showed enrichment of *Odoribacter*. No other treatment groups (LP11824, HK-LTJ53, HK-LP11824, PC) showed significant differences in microbial composition compared with the NC group in LEfSe analysis, suggesting that the LTJ53 strain had the most pronounced impact on gut microbiota structure under the current experimental conditions. Cluster heatmap analysis showed significant differences in bacterial species among the groups. At the phylum/class level, the model group showed enrichment of *Deferribacteres* (phylum) and *Saccharimonadia* (class), while the PC group mainly consisted of *Acidobacteriae*, *Fusobacteria*, *Ktedonobacteria*, *Halobacteria*, and *Cyanobacteriia*. In the LTJ53 group, we observed enrichment of *Gammaproteobacteria* and *Negativicutes*, which have been shown to improve the gut microecological environment by producing SCFAs. The HK-LTJ53 group was enriched with *Aanerolineae*, *Desulfobacteria*, and *Dissulfuribacter*. The dominant bacteria in the LP11824 group was *Myxococcia*, while the dominant bacteria in the HK-LP11824 group was *Acidimicrobiia*. Subsequently, based on the species annotation results at different taxonomic levels, the top 10 species with the highest abundance at the phylum level for each sample or group were selected to generate a cumulative relative abundance map of species. This allowed for a visual view of the species with higher relative abundance and their proportions at different taxonomic levels for each sample. *Proteobacteria* was enriched in the LTJ53 group. Previous studies have reported that certain members of this phylum can ferment polysaccharides and produce SCFAs; however, our sequencing resolution does not allow us to assign these functions to the specific OTUs enriched in this study. In the other intervention groups, we observed elevated levels of *Verrucomicrobiota*, which were enriched in each group; we hypothesize that this is related to mucin degradation, although further functional validation is required. In summary, through 16s sequencing analysis, we found that there were differences in the gut microbiota among the groups of mice. Each group affected the gut microbiota balance by influencing different microbiota, thus mediating the gut microecology.

### 3.6. L. plantarum and Its Postbiotic Regulate the Content of SCFAs in Constipated Mice

**Figure 6:**
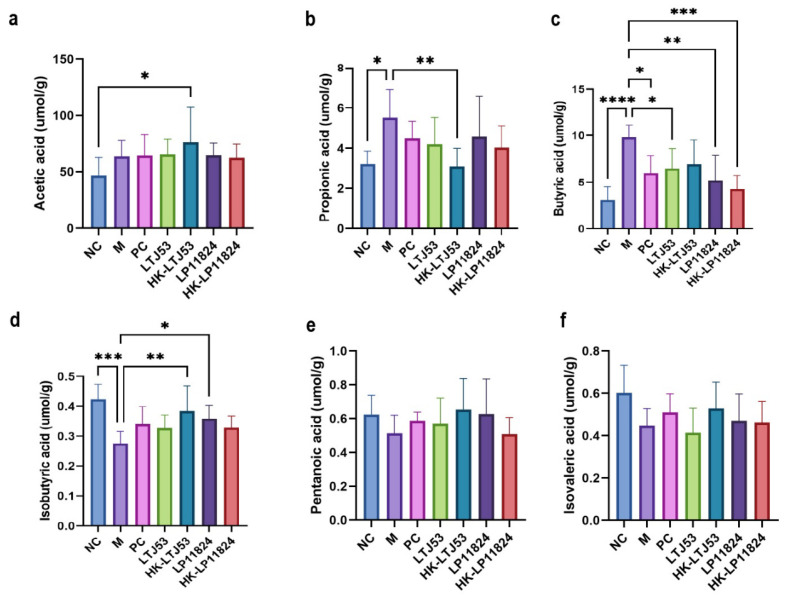
Effects on the production of SCFAs in constipated mice. (**a**) Acetic acid. (**b**) Propronic acid. (**c**) Butyric acid. (**d**) Pentanoic acid. (**e**) Isobutyric acid. (**f**) Isovaleric acid. Statistical significance: * *p* < 0.05, ** *p* < 0.01, *** *p* < 0.001, **** *p* < 0.0001 vs. M group. Data were log-transformed before ANOVA, FDR-corrected, n = 6.

SCFAs mainly include acetic acid, propionic acid, butyric acid, isobutyric acid, valeric acid, and isovaleric acid, which are produced by bacterial fermentation in the intestine into oligosaccharides, polysaccharides, polypeptides, proteins, and glycoproteins [25,26]. As shown in Figure 6, Acetic acid levels showed no significant statistical differences among most groups (Figure 6a). Notably, only the HK-LTJ53 group exhibited a significant increase in acetic acid content compared with the model group (*p* = 0.0352 < 0.05). The biological implication and strain-specific effect underlying this phenomenon remain unclear. A plausible explanation is that heat-inactivated LTJ53 retains structural components such as surface polysaccharides, which may specifically stimulate the growth and metabolic activity of acetate-producing bacteria. In addition, significantly higher levels of propionic acid (Figure 6b, *p* = 0.0149 < 0.05) and butyric acid (Figure 6c, *p* < 0.0001) were observed in the loperamide-treated model group compared with the normal group. Compared with the normal group, the model group had lower levels of isobutyric acid, valeric acid, and isovaleric acid, with a significant difference in isobutyric acid (*p* = 0.0002 < 0.001). The HK-LTJ53 group significantly increased the levels of acetic acid (*p* = 0.0352 < 0.05) and isobutyric acid (*p* = 0.0067 < 0.01) in the intestines of mice. In addition, we found an interesting phenomenon: in the constipated mouse model, the levels of propionic acid and butyric acid were significantly increased (*p* = 0.0149 < 0.05, *p* < 0.0001). One interpretation is that constipation prolongs colonic transit time, allowing prolonged fermentation of dietary fiber. However, an alternative interpretation is that loperamide directly or indirectly enriches butyrate- and propionate-producing taxa, independent of transit time. Notably, the beneficial effects of SCFAs on gut health are context-dependent; elevated SCFAs in the setting of constipation may reflect fermentative stasis rather than a functionally beneficial state. Therefore, the reduction in propionic and butyric acid levels after treatment (Figure 6b,c) should be interpreted as normalization of gut transit and fermentation dynamics, not as a loss of beneficial metabolites. After treatment, the intestinal throughput returned to normal. The shorter the time that dietary fiber stays in the colon, the less time it is fermented by bacteria. As a result, HK-LTJ53 significantly reduced the production of propionic acid (*p* = 0.0149 < 0.01), and the PC (*p* = 0.0118 < 0.05), LTJ53 (*p* = 0.0342 < 0.05), LP11824 (*p* = 0.002 < 0.01) and HK-LP11824 (*p* = 0.0002 < 0.001) groups all reduced the production of butyric acid. Elevated propionic and butyric acid levels in the model group may be explained by prolonged colonic transit time, which allows extended fermentation of dietary fiber by butyrate-producing bacteria. Alternatively, loperamide may directly affect SCFA production independent of transit time. These hypotheses require testing in follow-up experiments using controlled fermentation assays or transit-independent models.

### 3.7. Correlation Analysis of Constipation Biochemical Indicators, Gut Microbiota, and SCFAs in Constipated Mice

**Figure 7:**
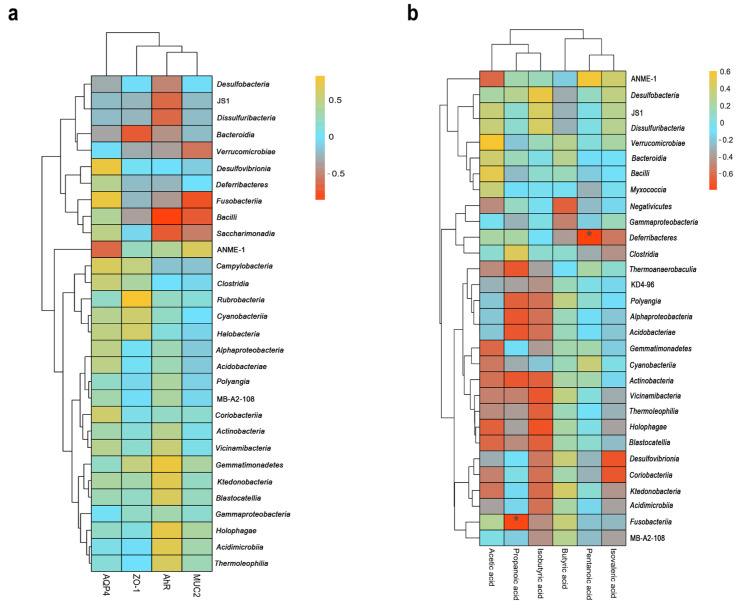
Sperman correlation analysis. (**a**) Correlation analysis between gut microbiota and constipation metabolic indicators. (**b**) Correlation analysis between gut microbiota and SCFAs. * *p* < 0.05 is considered statistically significant.

Through correlation clustering heatmap analysis of the abundance of gut microbiota, metabolic indicators, and SCFAs in constipated mice, we observed a series of significant associations between different bacterial groups and host metabolites and immune signaling pathways [27]. This further reveals the potential regulatory role of gut microbiota in the occurrence and development of constipation. The results are shown in Figure 7. It is worth noting that, in terms of the direction and strength of the correlation, many bacterial groups showed significant positive or negative associations with key metabolites and signaling molecules. *Fusobacteria* abundance was negatively correlated with valeric acid levels. This correlation generates a hypothesis that this taxon might influence valeric acid metabolism, but causality cannot be inferred, and functional assignment at this taxonomic level is inherently limited; meanwhile, valeric acid was significantly negatively correlated with *Deferribacteres*, indicating that this group may participate in intestinal dysfunction by affecting valeric acid metabolism in constipation. Furthermore, the observed correlations among *Gemmatimonadetes*, acetic acid, and AhR suggest a possible interplay; however, the direction and nature of these relationships require experimental validation. Regarding host immune metabolic signaling, *Dissulfuribacter* showed a significant negative correlation with AhR, suggesting that this type of sulfur-metabolizing bacteria may participate in the constipation process by affecting the activity of the AhR pathway. The positive correlation between *Gemmatimonadetes* and AhR further supports the close link between specific bacterial community structures and abnormal AhR signaling. These associations collectively indicate that, in a state of constipation, the dysbiosis of the gut microbiota not only affects the synthesis and metabolism of SCFAs, but may also further exacerbate intestinal motility dysfunction and local immune metabolic disorders by regulating key host signaling pathways such as AhR. The correlation analysis in this figure is based on Spearman rank correlation coefficient. Statistical significance does not imply biological correlation or causality.

## 4. Discussion

Constipation is a common gastrointestinal disease that seriously affects patients’ quality of life, and current treatments are often limited by side effects or drug dependence [28]. This study investigated the efficacy of *L. plantarum* LTJ53 and 11824 and their postbiotics in relieving loperamide-induced constipation in mice.

**Figure 8:**
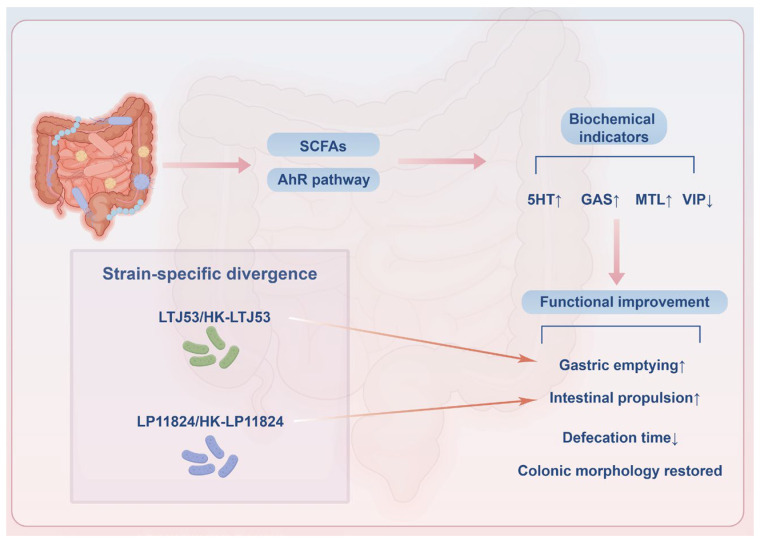
Proposed integrated mechanism of *L. plantarum* and its postbiotics in relieving constipation. The figure summarizes the multi-step cascade: (1) microbiota remodeling, (2) SCFA modulation and AhR activation, (3) neurotransmitter/hormone regulation, and (4) functional improvement. Dashed arrows indicate strain-specific divergence between LTJ53 and LP11824. ↑ indicates upregulation, ↓ indicates downregulation. Thanks to Figdraw 2.0.

Rather than presenting each finding independently, we integrate them into a coherent multi-step model linking microbiota remodeling to symptom relief. Briefly, we propose that *L. plantarum* and its postbiotics reshape the gut microbiota (Step 1), which alters SCFAs profiles and activates AhR signaling (Step 2). These changes, in turn, modulate enteroendocrine and neuronal pathways, increasing prokinetic neurotransmitters (5-HT, GAS, MTL) while suppressing inhibitory VIP (Step 3). The net effect is accelerated gastric emptying and intestinal transit, along with restored colonic histology (Step 4). The following sections elaborate on each step and the strain-specific divergences observed (Figure 8).

First, treatment with two strains of *L. plantarum* and their postbiotics significantly improved weight gain, gastric emptying, and small intestinal motility in constipated mice. These physiological improvements are accompanied by increased levels of 5-HT, GAS, and MTL, as well as decreased VIP secretion. 5-HT is a key neurotransmitter in the gut–brain axis, stimulating intestinal motility and secretion [29]. Similarly, GAS and MTL are known to promote gastric emptying and intestinal motility, and their recovery in treated mice further supports the prokinetic effects of *L. plantarum* and its postbiotics [30]. Conversely, the inhibitory neurotransmitter VIP was significantly reduced in all treatment groups, indicating a shift in motor activity towards excitatory activity [31]. Histological examination showed that both live *L. plantarum* and its postbiotics could restore the integrity of colonic tissue and increase the thickness of the muscle layer. These morphological improvements are consistent with the upregulation of tight junction proteins ZO-1 and mucin MUC2, both of which are essential for maintaining intestinal barrier function and lubrication. Notably, the aquaporin AQP4, associated with excessive water absorption during constipation, was downregulated in all treatment groups, indicating reduced water reabsorption and improved fecal water content. The AhR pathway, which plays a role in immune regulation and gut homeostasis, was also upregulated, suggesting that *L. plantarum* and its postbiotics may regulate host physiology through this pathway [32].

Correlation analysis further highlighted the interactions among gut microbiota, SCFAs, and host metabolic indicators. These correlations are consistent with the hypothesis that specific bacterial groups could mediate host physiological effects through metabolic signaling. Alternative explanations cannot be ruled out. *Fusobacteria* was negatively correlated with propionic acid, while *Gemmatimonadetes* was positively correlated with AhR and negatively correlated with acetic acid. These associations suggest that specific bacterial groups may mediate the effects of *L. plantarum* on host physiology through metabolic signaling and immune regulation. The elevated levels of propionic and butyric acid in the model group can also be explained by changes in gut microbiota composition. As shown in Figure 5, the model group was enriched in *Deferribacteres* and *Saccharimonadia*, and these taxa may contain members capable of producing butyric and propionic acid. Consistent with this hypothesis, Spearman correlation analysis (Figure 7b) revealed that butyric acid was significantly positively correlated with *Fusobacteria*. Therefore, it is reasonable that loperamide-induced constipation reshapes the gut microbiota toward a community with enhanced SCFA-producing capacity, independent of intestinal transit time. Accordingly, the reduction in SCFAs levels after treatment may reflect the restoration of a more balanced microbial community, rather than a direct negative effect on SCFA production. However, 16S rRNA-based functional inference can only indicate associations, not causation. It is worth noting that LTJ53 and LP11824 showed different effects in improving constipation. LTJ53 was more effective than LP11824 in improving weight loss, gastric emptying rate, and related metabolic indicators, while LP11824 was better in terms of black stool passage time and small intestinal propulsion rate. In summary, LTJ53 and its postbiotic showed stronger performance in overall metabolic recovery and neuroimmune regulation, and were suitable for systemic constipation intervention, while LP11824 had an advantage in promoting intestinal peristalsis and was suitable for constipation types that were mainly slow.

Several limitations should be acknowledged. First, the loperamide-induced model mimics drug-acute constipation but not chronic functional constipation in humans. Second, treatment duration was limited (14 days), and long-term efficacy/safety were not assessed. Third, 16S rRNA sequencing data can only reveal correlations, not causality; mechanistic studies are needed. Fourth, formal safety assessments, formulation stability tests, and dose–response analyses were not performed. Thus, statements on safety, stability, or clinical applicability remain preliminary. Fifth, the sample size (n = 6/group) is adequate for large effect sizes but may underpower small-to-moderate effects; independent replication is warranted.

Based on this study on the difference in efficacy between two strains, LTJ53 and 11824, in improving constipation, it is suggested that the probiotic industry should establish a strain-specific functional labeling system and develop precise intervention products targeting different constipation subtypes [33,34,35].

## 5. Conclusions

This study shows that live *L. plantarum* LTJ53 and 11824, along with their heat-inactivated postbiotics, can effectively alleviate loperamide-induced constipation in mice by regulating gut microbiota structure, enhancing gastrointestinal motility, improving intestinal barrier function, and modulating the levels of neurotransmitters related to the brain–gut axis. The two strains showed strain specificity in improving constipation: LTJ53 was more effective in restoring weight and gastric emptying, while LP11824 was more effective in promoting small intestinal propulsion.

## Figures and Tables

**Table 1:** The primer sequence for RT-PCR.

Gene	Primer Pair	Primer Sequence (5′-3′)
GAPDH	Forward	AACAGCAACTCCCACTCTTC
	Reverse	CCTGTTGCTGTAGCCGTATT
AQP4	Forward	TGGGCAAACCACTGGATATATTG
	Reverse	GTCTTCCGTCTCCACTTGGC
AhR	Forward	TTGGTTGTGATGCCAAAGGG
	Reverse	CTCCAGCGACTGTGTTTTGC
ZO-1	Forward	AAGATGGGATTCTTAGGCCCAGCA
	Reverse	TCTTTGGCTGCAGGGCTATCTTCT
MUC2	Forward	GTCCTGACCAAGAGCGAACA
	Reverse	ACAGCACGACAGTCTTCAGG

## Data Availability

The data presented in this study are available within the article.

## Abbreviations

The following abbreviations are used in this manuscript:

5-HT	5-hydroxytryptamine
SCFA	Short-chain fatty acid
GAS	Gastrin
MTL	Motilin
VIP	Vasoactive intestinal peptide
ZO-1	Zonula Occludens-1
MUC2	Mucin 2
AhR	Aryl hydrocarbon receptor
AQP4	Aquaporin 4
GABA	γ-aminobutyric acid
GAPDH	Glyceraldehyde-3-phosphate dehydrogenase
PCoA	Principal Coordinates Analysis
